# The role of autophagy in the cytotoxicity induced by trastuzumab emtansine (T-DM1) in HER2-positive breast cancer cells

**DOI:** 10.1186/s13568-020-01044-0

**Published:** 2020-06-03

**Authors:** Peipei Liu, Jiajun Fan, Ziyu Wang, Wenjing Zai, Ping Song, Yongping Li, Dianwen Ju

**Affiliations:** 1 Department of Biological Medicines & Shanghai Engineering Research Center of Immunotherapeutics, Fudan University School of Pharmacy, Shanghai, 201203 China; 2 Department of Pharmacy, Huadong Hospital, Fudan University, Shanghai, 200040 China; 3 School of Basic Medical Science, Fudan University, Shanghai, 201203 China; 4 Department of Pharmacy, Ruijin Hospital Luwan Branch, School of Medicine, Shanghai Jiao Tong University, Shanghai, 200020 China; 5 Department of Breast Surgery, Shanghai Pudong Hospital, Fudan University Pudong Medical Center, Shanghai, 201399 China

**Keywords:** Trastuzumab emtansine, Autophagy, Apoptosis, Autophagy inhibition

## Abstract

Trastuzumab emtansine (T-DM1), an antibody–drug conjugate (ADC) of trastuzumab and cytotoxic agent emtansine (DM1), has been approved for the therapy of metastatic HER2-positive breast cancer after prior treatment of trastuzumab and taxane. The impressive efficacy exhibited by T-DM1 has heightened the need for more further studies on the underlying mechanisms of T-DM1 cytotoxicity. Previous research suggested that autophagy was crucial for cancer therapy, but the role of autophagy in T-DM1 treatment has not been investigated. Here, we demonstrated for the first time that T-DM1 triggered obvious autophagy in HER2-positive SK-BR-3 and BT-474 breast cancer cells. Blocking autophagy with pharmacological inhibitors chloroquine (CQ) or LY294002 partly reduced T-DM1-induced apoptosis and Caspase-3/7 activation, suggesting that autophagy played an essential role in the cytotoxicity induced by T-DM1 in HER2-positive breast cancer cells. Further investigation demonstrated that Akt/mTOR signaling pathway was involved in T-DM1-induced autophagy in a time-dependent manner. Altogether, our results highlighted the important role of autophagy as a novel mechanism for T-DM1-induced cytotoxicity and elucidated the critical relationships between T-DM1-induced autophagy and apoptosis in human HER2-positive breast cancer cells, which provides novel insight into the underlying anti-tumor mechanism of T-DM1.

## Key points

- T-DM1 induced apoptosis in HER2-positive breast cancer cells.
- T-DM1 triggered obvious autophagy in HER2-positive breast cancer cells.
- Inhibiting autophagy attenuated T-DM1-induced apoptosis and cytotoxicity in HER2-positive breast cancer SK-BR-3 and BT-474 cells.
- T-DM1 blocked Akt/mTOR signaling pathway activation in HER2-positive breast cancer cells.

## Introduction

Approximately 20–30% patients with breast cancer are characterized with overexpressed human epidermal growth factor receptor 2 (HER2) which is closely related with short time of recurrence, high risk of metastases and poor overall patient survival (Loibl and Gianni 2017; Rimawi et al. 2015). Trastuzumab, a humanized monoclonal antibody which targets HER2 extracellular domain, has been approved since 1998 as the prior neoadjuvant treatment for HER2-overexpressed metastatic breast cancer (Romond et al. 2005; Slamon et al. 2011). In spite of the improvements in outcomes of HER2-targeted therapy, approximately a quarter of patients who have received HER2-targeted therapy plus neoadjuvant chemotherapy are then found to have residual invasive breast cancer at surgery (Darini et al. 2019; Zhang et al. 2011). These limitations raise the necessity for achieving better understanding of biological mechanisms of these agents and developing better approaches for the treatment of HER2-overexpressed breast cancer.

Trastuzumab emtansine (T-DM1) is a HER2-targeted antibody–drug conjugate (ADC) which covalently binds trastuzumab with toxic agent emtansine (DM1), a potent microtubule inhibitor, by non-reducible thioether linker (Hurvitz et al. 2018). T-DM1 was approved for the therapy of HER2-overexpressed metastatic breast cancer in patients who have progressed despite previously receiving therapy of trastuzumab and taxane (Hunter et al. 2020). T-DM1 retains trastuzumab activity and it can deliver DM1 to HER2-positive cells efficiently. T-DM1 exhibits superior efficacy than trastuzumab mono-treatment or trastuzumab combined with other chemotherapeutic agents (Chen et al. 2016; Singh et al. 2016). Mechanistically, T-DM1 is internalized into the intracellular compartment upon interacting with HER2 receptor on cell surface, and then emtansine is degraded by proteases of acid lysosomes to release cytotoxic metabolites that act on tubulin (Erickson et al. 2006). Although T-DM1 has yielded impressive efficacy in the clinic, resistance, relapse and systemic toxicity of this targeted therapy can still arise (Rios-Luci et al. 2017). Thus, it’s crucial to conduct in-depth investigation on the molecular mechanisms of T-DM1, which could then provide novel strategies to optimize clinical outcomes.

Autophagy is an evolutionarily conserved mechanism that mediates dysfunctional protein and organism degradation (Cao et al. 2019). Growing evidence shows that autophagy plays dual roles in tumorigenesis and cancer therapy (Gewirtz 2013; Yun and Lee 2018). Many anti-tumor agents including imatinib and proteasome inhibitors have been reported to induce cytoprotective autophagy which is partly responsible for drug resistance (Zeng et al. 2015; Zhu et al. 2010). However, autophagy is also recognized as a novel kind of programmed cell death in certain circumstance. Poly(amidoamine) dendrimers led to robust cell death and tissue damage by triggering cytotoxic autophagy (Li et al. 2014). Lapatinib, an oral dual tyrosine kinase inhibitor, was reported to induce autophagy, which facilitated apoptosis in HER2-positive breast cancer (Zhu et al. 2013). Recently, autophagic cell death was also observed in non-Hodgkin lymphoma which was exposed to Rituximab-monomethyl auristatin E, indicating a possible interplay between ADC and autophagy (Wang et al. 2018). However, whether T-DM1 could induce autophagy in cancer treatment has not been defined, nor has the relationship between autophagy and T-DM1 efficacy been determined.

This paper attempts to determine whether T-DM1 could trigger autophagy in HER2-overexpressed breast cancer cells. Our data showed that T-DM1 induced obvious autophagy in SK-BR-3 and BT-474 cells and blocking autophagy partly reversed cytotoxicity and apoptosis induced by T-DM1, suggesting autophagy played a vital part in the cytotoxicity induced by T-DM1 in HER2-positive breast cancer cells. Deciphering role of autophagy as well as underlying signaling mechanism of T-DM1 could contribute to further understanding of T-DM1 resistance and provide novel idea for the design of second-generation HER2-targeted ADC.

## Materials and methods

### Regents

T-DM1 was purchased from Genentech Roche (San Francisco, CA, USA). Apoptosis Detection Kit was from Dojindo (Tokyo, JPN). Z-VAD-fmk, rapamycin, LY294002 and chloroquine (CQ) were obtained from Sigma-Aldrich (St Louis, MO, USA).

### Cell culture

HER2-positive human breast cancer cell lines BT-474 as well as SK-BR-3 were from American Type Culture Collection (Rockville, MD, USA). Both two cells were cultured in RIPM-1640 medium supplemented with 10% fetal bovine serum.

### Cell viability analysis

Cell counting kit-8 (CCK-8) (Dojindo, Tokyo, JPN) was applied for cell viability measurement. At first, cells were cultured with indicated doses of T-DM1 and/or autophagy inhibitors. After 3 days, CCK-8 solution was carefully transferred to plates and the samples were then maintained at 37 °C for another 3 h. Cell viability was analyzed and expressed as percentage of control absorbance.

### Transmission electron microscopy (TEM)

Cells were exposed to T-DM1 for 72 h, then harvested and processed as previously described (Zeng et al. 2015). The ultra-structure morphology was visualized via TEM (JEOL, Inc., USA).

### Western blot

After treatment, cells were lysed and incubated on ice for half an hour, and then supernatant was obtained and quantified. Equivalent amount protein was separated using SDS-PAGE and transformed on polyvinylidene fluoride membrane that was blocked by 5% bovine serum albumin for 60 min afterwards. Next, membranes were exposed to specialized antibodies and secondary antibodies (Cell Signaling Technology, MA, USA). Finally, samples were examined by enhanced chemiluminescence reagents (Millipore, Billerica, MA, USA).

### Confocal microscopy

Rapamycin (50 nM) was employed as a positive control. After treatment, samples were cultured with Cyto-ID Green dye (ENZO Life Science, NY, USA) following the manufacturer’s protocol. Finally, samples were examined by confocal microscope (LSM710, Carl Zeiss, Germany).

### Caspase-3/7 activity analysis

For the measurement of apoptosis, cells were exposed to T-DM1 for 48 h and cultured with Caspase-Glo 3/7 reagent (Promega, Madison, USA) for half an hour at 37 °C. Caspase-3/7 activity was then determined by microplate reader (SpectraMax M5, Molecular Devices, USA).

### Statistical analysis

Statistical analysis was performed by Students’ *t*-test (two-tailed) or One-Way analysis via GraphPad Prim 5, and the data of three independent experiments were expressed as mean ± standard deviations (SD) (*P < 0.05, **P < 0.01, ***P < 0.001).

## Results

### T-DM1 induced apoptosis in SK-BR-3 and BT-474 cells

**Fig. 1:**
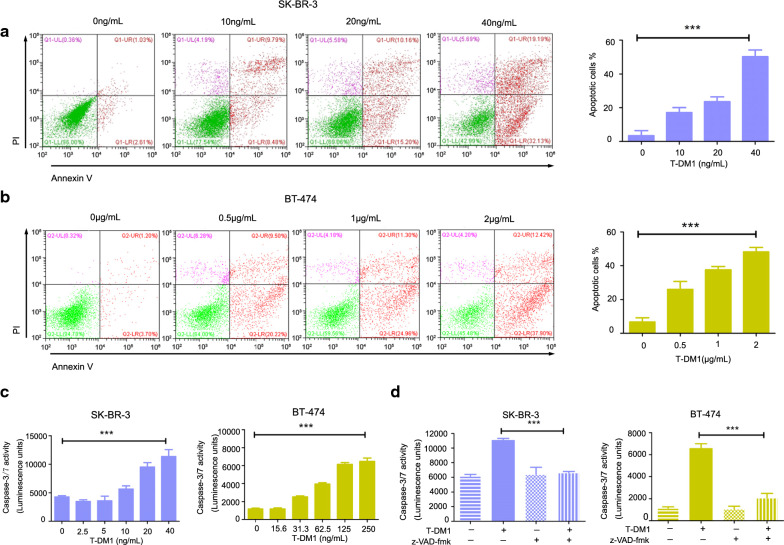
T-DM1 induced apoptosis and Caspase-3/7 activation in SK-BR-3 and BT-474 cells. **a**, **b** Cells were incubated with T-DM1 for 2 days, and then examined by flow cytometry. Percentages of Annexin V/PI-positive cells were shown in bar charts. **c** Cells were cultured with different concentrations of T-DM1 for 2 days and caspase-3/7 activation was analyzed by commercial kits. **d** SK-BR-3 cells (20 ng/mL of T-DM1) and BT-474 cells (250 ng/mL of T-DM1) were exposed to 20 μM of Z-VAD-fmk for 2 days and Caspase-3/7 activation of both two cells was detected

For evaluating anti-tumor effect of T-DM1 on HER2-overexpressed breast cancer cells, flow cytometry was used to detect Annexin V/PI-positive cells. As shown in Fig. 1a, b, there was an obvious increase of Annexin V/PI-positive cells in a dose-dependent manner in SK-BR-3 and BT-474 cells after T-DM1 treatment for 2 days, indicating that T-DM1 induced potent apoptosis in both two breast cancer cells. Specifically, SK-BR-3 cells were more sensitive to T-DM1 than BT-474 cells, which was consistent with HER2 expression levels in these two cells as reported in previous studies (Lewis Phillips et al. 2008; Takegawa et al. 2019). Caspase-3/7 activation is the key biomarker of apoptosis. As illustrated in Fig. 1c, T-DM1 triggered a significant up-regulation of Caspase-3/7 activation level in the cellular lysate of SK-BR-3 and BT-474 cells. Moreover, blocking apoptosis by Z-VAD-fmk, a general caspase-family inhibitor, significantly attenuated T-DM1-mediated Caspase-3/7 activation (Fig. 1d).

Collectively, our results suggested that T-DM1 could induce significant apoptosis in HER2-positive breast cancer cells.

### Autophagy was triggered by T-DM1 in HER2-positive breast cancer cells

**Fig. 2:**
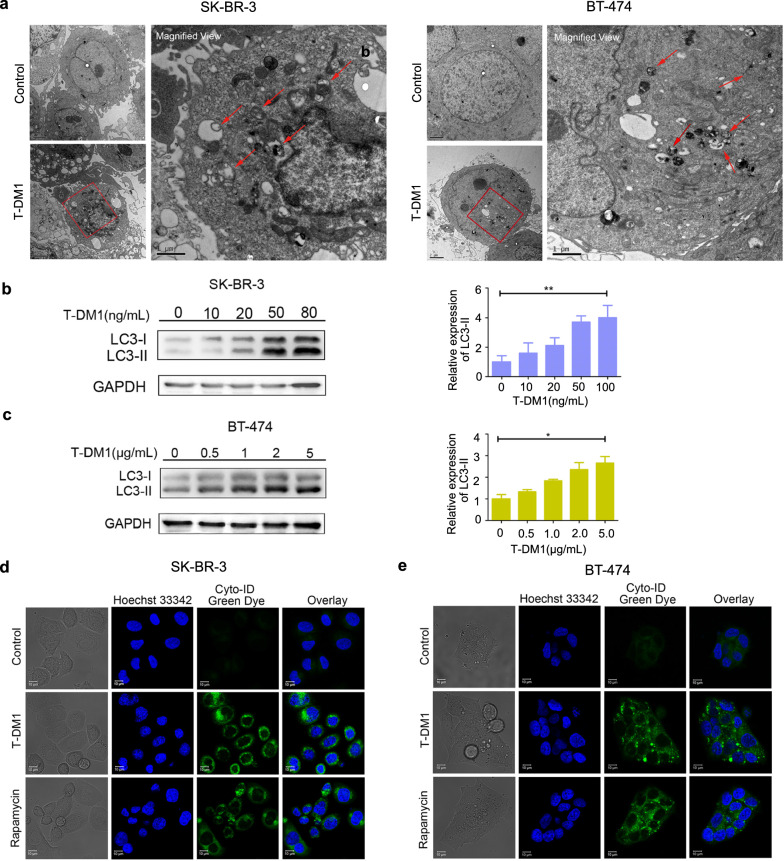
Autophagy was triggered by T-DM1 in SK-BR-3 and BT-474 cells. SK-BR-3 cells were treated with 20 ng/mL of T-DM1 and BT-474 cells were cultured with 500 ng/mL of T-DM1 for 3 days. **a** Representative electron micrographs of T-DM1-treated SK-BR-3 as well as BT-474 cells. **b**, **c** LC3-I/II expression levels were examined after T-DM1 treatment. Relative expression levels of LC3-II/GAPDH were presented in bar charts. **d**, **e** Accumulations of autophagosomes in breast cancer cells were observed with Cyto-ID staining by confocal microscopy

Considering that autophagy could be triggered under stress conditions and plays a crucial role in anti-cancer therapy, we then investigated whether T-DM1 could induce autophagy. First, ultra-structure morphologic analysis was applied to detect the formation of autophagosomes by transmission electron microscopy. As illustrated in Fig. 2a, an obvious accumulation of “double-membrane” autophagic vesicles can be found in the cytoplasm of these two HER2-positive breast cancer cells. To further confirm the formation of autophagy, LC3-I/II, the autophagy-related protein, was determined by western blot. Figure 2b, c showed that LC3-II expression levels significantly increased as compared with normal cells in dose-dependent manners after T-DM1 treatment. Finally, we employed Cyto-ID^®^ autophagy green dye to detect autophagosomes and assess autophagy level changes by confocal microscopy. As displayed in Fig. 2d, e, T-DM1 treatment induced a remarkable autophagic fluorescent accumulation in the cytoplasm of these two HER2-positive cells. Rapamycin, an autophagy inducer, served as a positive control.

Overall, the data indicated T-DM1 triggered autophagy in both two HER2-overexpressed breast cancer cells.

### Autophagy inhibition reversed T-DM1-indcued cytotoxicity and apoptosis in HER2-positive breast cancer cells

**Fig. 3:**
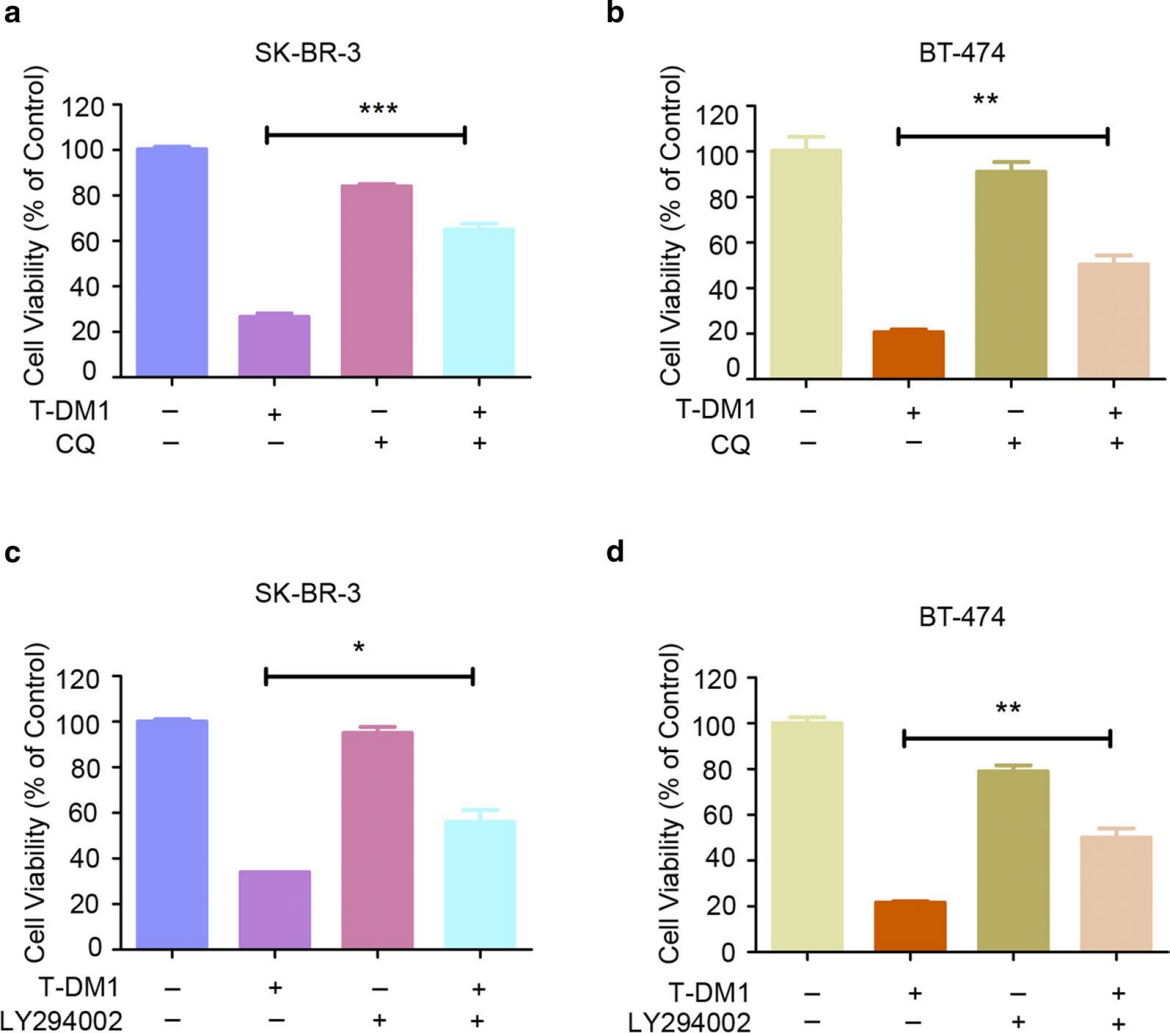
Autophagy inhibition reversed T-DM1-induced cytotoxicity in both two HER2-positive cells. **a**, **b** Before CCK-8 assay, the breast cancer cells were cultured with indicated concentrations of T-DM1 combined with CQ (10 μM) for 3 days. **c**, **d** Before cell viability analysis, cells were treated with corresponding concentrations of T-DM1 with or without of 5 μM of LY294002 for 3 days

Given that T-DM1 induced autophagy in these two HER2-positive breast cancer cells, we wondered whether autophagy played a pro-survival role or cytotoxic role in T-DM1-mediated anti-tumor efficacy. To test the actual roles of autophagy, we treated both HER2-positive breast cancer cells with T-DM1 and CQ, which was a typical inhibitor that inhibited the integrating of lysosomes and autophagosomes and blocked autophagosome formation. Our results (Fig. 3a, b) showed that T-DM1 combined with CQ treatment partly reversed T-DM1-induced cytotoxocity in these two breast cancer cells. In accord with the findings of CQ above, another autophagy inhibitor LY294002 also partly rescued these two HER2-positive breast cancer cells from T-DM1-induced cytotoxicity (Fig. 3c, d).

**Fig. 4:**
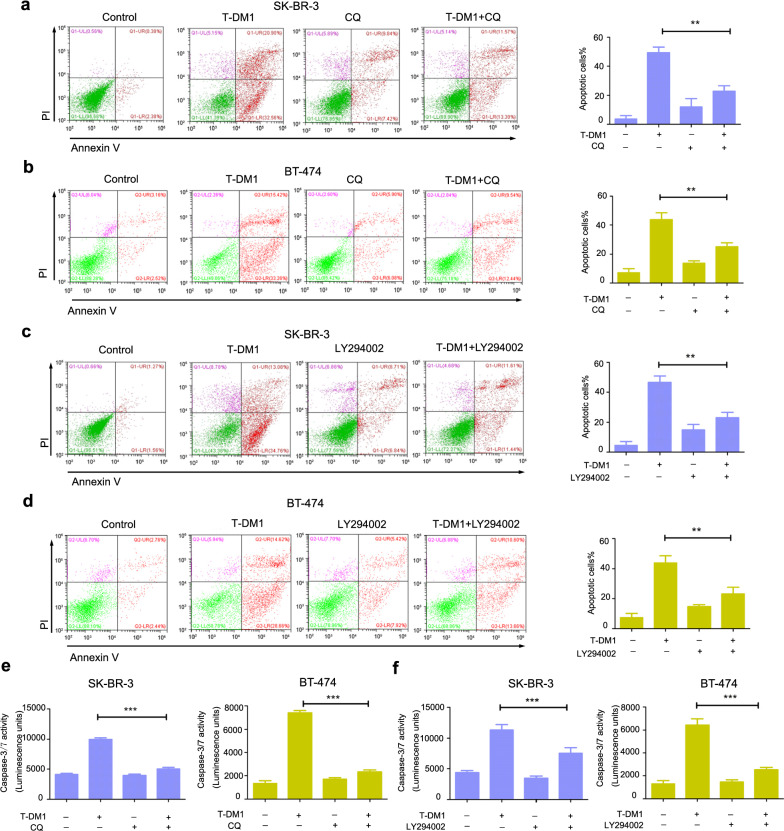
Inhibition of autophagy reduced T-DM1-induced apoptosis in SK-BR-3 cells and BT-474 cells. SK-BR-3 cells and BT-474 cells are exposed to corresponding concentrations of T-DM1 with or without 5 μM of LY294002 or 10 μM of CQ for 2 days. **a**–**d** Proportion of Annexin V/PI-positive cells were analyzed and then presented in bar charts. **e**, **f** After treatment with T-DM1 in combination with autophagy inhibitors, the activation of caspase-3/7 was analyzed by commercial kit and presented in bar charts

Moreover, we also studied the role of autophagy in T-DM1-induced apoptosis in these two breast cancer cells. As presented in Fig. 4a, b, T-DM1 treatment alone induced significant cellular apoptosis in both HER2-positive breast cancer cells, whereas combinational treatment of T-DM1 and CQ drastically reduced T-DM1-medicated apoptotic cell death. Meanwhile, the results of LY294002 were in accord with CQ in both breast cancer cells (Fig. 4c, d). Caspase-3/7 analysis also revealed that inhibiting autophagy by CQ or LY294002 remarkably reduced T-DM1-induced Caspase-3/7 activation level (Fig. 4e, f).

Altogether, blocking autophagy by chemical inhibitors significantly reversed T-DM1-medicated apoptosis, indicating that autophagy induced by T-DM1 played a cytotoxic role facilitating apoptosis in these two cells.

### Akt/mTOR signaling pathway inactivation was associated with T-DM1-induced autophagy in HER2-overexpressed breast cancer cells

**Fig. 5:**
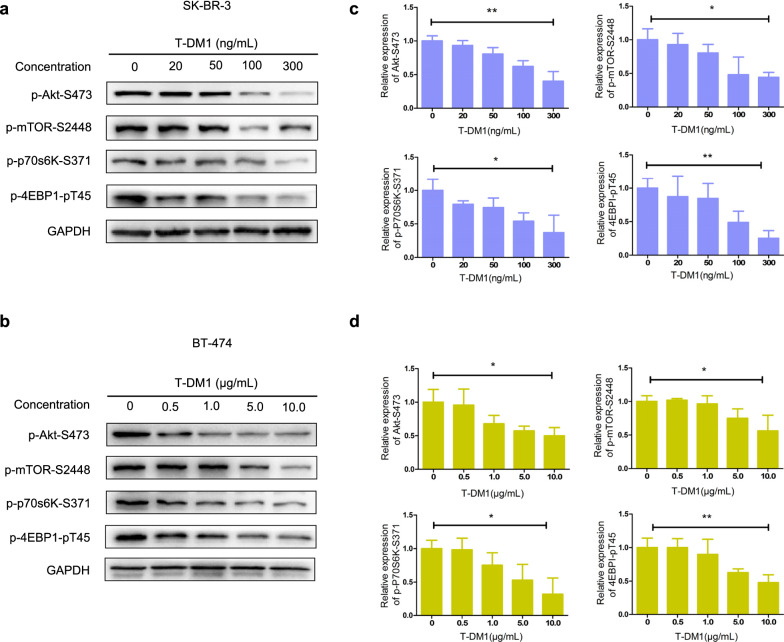
Akt/mTOR signaling pathways were associated with T-DM1-induced autophagy in both two cells. **a** SK-BR-3 cells were incubated with 0, 20, 50, 100 and 300 ng/mL of T-DM1 for 48 h, and the protein expression levels of p-mTOR-S2448, p-p70s6K-S371, p-4EBP1-pT45, Akt and p-Akt-S473 were determined by western blot analysis. **b** Relative protein expression levels compared to GAPDH were showed in bar charts. **c** Protein expression levels were determined by western blot, after BT-474 cells being cultured with indicated concentrations of T-DM1 for 2 days. **d** Relative protein expression levels of BT-474 cells were presented in bar charts

Previous studies showed that Akt/mTOR pathway which functions via sensing intracellular energy stress was one of the major regulators of autophagy (Xia et al. 2016). To explore the molecular mechanisms of T-DM1-induced autophagy, we examined the levels of mTOR phosphorylation and its subsequent transductions. As exhibited in Fig. 5a, b, T-DM1 significantly reduced mTOR phosphorylation in dose-dependent manners in both two HER2-positive breast cancer cells. 4E-binding protein 1 (4EBP1) and Protein S6 kinase (p70s6K), downstream components of mTOR, were also significantly dephosphorylated. Moreover, the Akt phosphorylation level was also remarkably reduced upon T-DM1 treatment dose-dependently. The quantitative analyses of proteins mentioned above were shown in Fig. 5c, d.

Collectively, these results showed T-DM1 could inhibit Akt/mTOR signaling pathway and Akt/mTOR pathway inactivation was associated with T-DM1-triggered autophagy in both HER2-positive breast cancer cells.

## Discussion

Since the identification of *HER2* gene was considered as an important oncological driver of a subset of breast cancers, the development of HER2-based therapy has achieved advanced progression. The therapeutics consists of tyrosine kinase inhibitors, humanized monoclonal antibodies and ADC (Rinnerthaler et al. 2019). T-DM1, which covalently links trastuzumab with maytansinoid derivative DM1, has exhibited excellent efficacy in the clinic (Peters et al. 2019). T-DM1 retains original activities of trastuzumab including recognizing HER2 on the cellular surface of breast tumor and down-regulating the subsequent PI3K-AKT signaling pathway. Antibody-dependent cell-mediated cytotoxicity (ADCC) is also maintained in T-DM1 therapy (Krop and Winer 2014). Because T-DM1 can selectively deliver DM1 to HER2-overexpressed malignant cells, the exposure of cell-killing DM1 to systemic tissues is significantly reduced and the therapeutic window of this agent is thus improved (Junttila et al. 2011). Despite the concept of T-DM1 design is straightforward and the efficacy of T-DM1 has been well-validated in the clinic, underlying function mechanism of this agent has not been completely elucidated yet.

This report attempts to uncover underlying mechanisms of T-DM1 that lead to cell death in human HER2-overexpressed breast cancer cells. First, we demonstrated that apoptosis was triggered upon T-DM1 treatment in two HER2-positive breast cancer cells. Moreover, T-DM1-induced apoptosis was showed to be Caspase-3/7-dependent, blocking apoptotic cell death via molecular agent Z-VAD-fmk partly reversed T-DM1-mediated cytotoxicity and Caspase-3/7 activation. These results indicated that there might be other types of cell death aside from apoptosis that participated in T-DM1-induced anticancer effects.

Mounting evidence shows that autophagy which plays an important role in anti-tumor treatment. While autophagy majorly serves as a protective role for drug resistance, it is also recognized as type II programmed cell death and mediates chemotherapeutics toxicity in certain cases (Chen et al. 2012; O’Donovan et al. 2011; Yang et al. 2011). Recently, autophagy has been identified to be involved in Rituximab-MMAE-induced anti-tumor efficacy in non-Hodgkin lymphoma, indicating a cytotoxic role of autophagy in Rituximab-MMAE-based tumor therapy (Wang et al. 2018). In this essay, it is for the first time that we demonstrated that T-DM1 significantly triggered autophagy in HER2-positive breast cancer cells, as evidenced by confocal microscopy, transmission electron microscopy and western blot analysis. Specifically, blocking autophagy by pharmacological inhibitors including CQ and LY294002 partly reversed T-DM1-induced cytotoxicity, indicating a cytotoxic role of autophagy in T-DM1 treatment. Remarkably, CQ and LY294002 partly reversed T-DM1-induced apoptotic cell death and reduced Caspase-3/7 activation. These results elucidated that there was a close crosstalk between T-DM1-induced apoptosis and autophagy, and the cytotoxic autophagy facilitated apoptotic cell death in T-DM1 treatment. Further study on the relationship of autophagy and apoptosis in T-DM1 therapy is meaningful to develop novel therapeutic strategies for improving the therapeutic efficacy.

To elucidate molecular mechanism of T-DM1-induced autophagy, we investigated Akt/mTOR signaling pathway, which negatively modulates autophagy. Our results demonstrated that T-DM1 treatment significantly reduced p-mTOR-S2448 expression levels in both two cells. The subsequent regulators of mTOR, including p70s6K and 4EBP1, were also significantly dephosphorylated in dose-dependent manners. Besides, Akt, an upstream regulator of mTOR, was significantly dephosphorylated upon T-DM1 treatment. The results mentioned above showed that Akt/mTOR pathway was highly involved in T-DM1-induced autophagy in human HER2-overexpressed breast cancer cells.

**Scheme 1:**
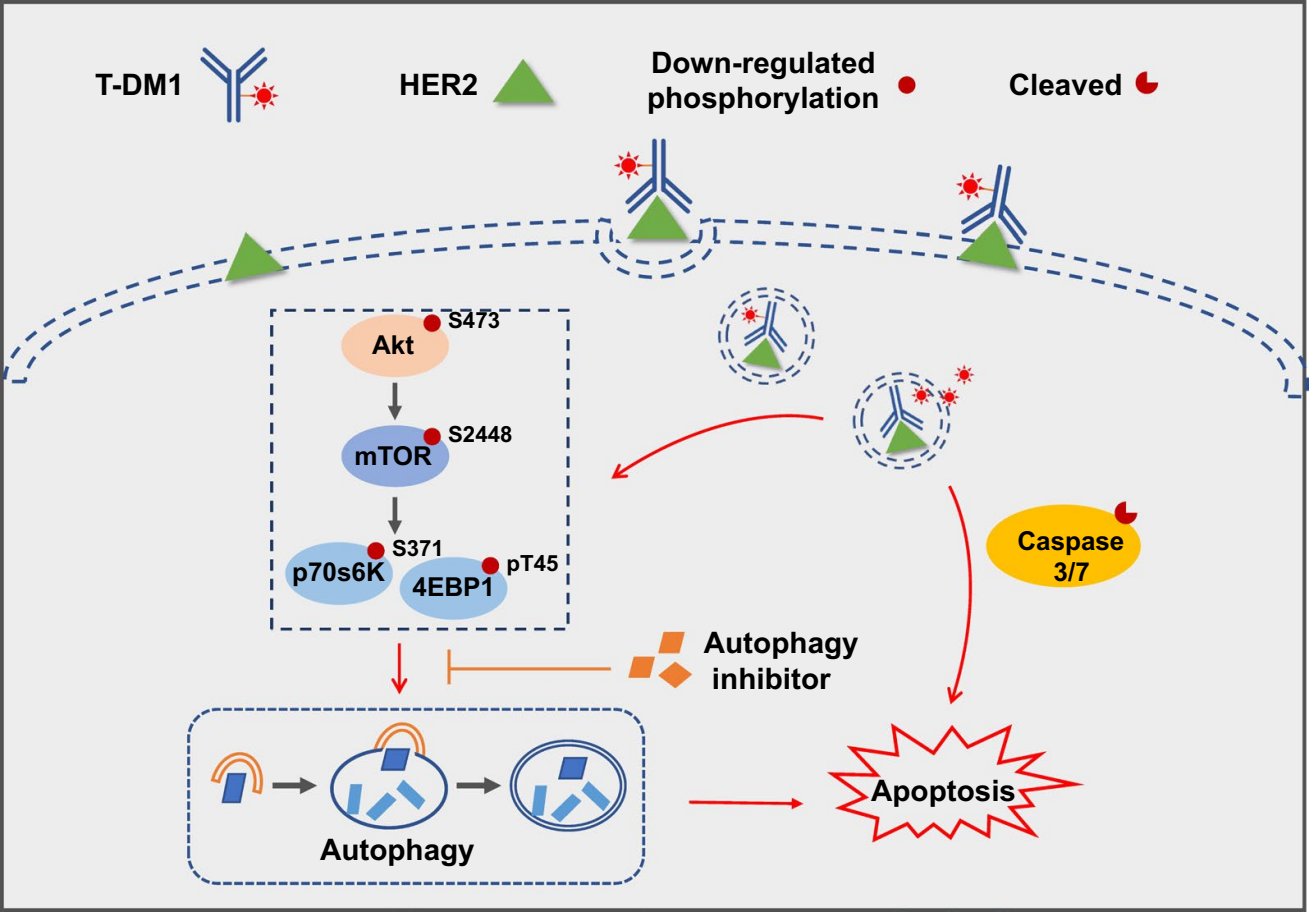
Schematic illustration of the role of autophagy in T-DM1-triggered cytotoxicity in SK-BR-3 as well as BT-474 cells. In brief, T-DM1 is internalized upon interacting with HER2 on the cellular surface, and degraded into cytotoxic metabolites by proteases in lysosomes. The toxic agent promotes Caspase-3/7 dependent apoptosis and autophagy afterwards. Mechanistically, Akt/mTOR pathway inhibition is crucial in autophagy triggered by T-DM1, and the relationship between autophagy and apoptosis is also identified. Blocking autophagy via pharmacological inhibitors partly reduced T-DM1-induced apoptotic cell death, indicating that autophagy triggered by T-DM1 therapy played a cytotoxic role which facilitated apoptosis in human HER2-overexpressed breast cancer cells

In conclusion, it was for the first time we demonstrated that T-DM1, a clinical approved ADC agent, could trigger autophagy in the two HER2-overexpressed breast cancer cells. Scheme 1 illustrates the role of autophagy in the cytotoxicity induced by T-DM1 in HER2-overexpressed breast cancer cells. Inhibiting autophagy by pharmacological inhibitors (CQ and LY294002) partly reduced T-DM1-induced cytotoxicity and apoptotic cell death, indicating a close interaction between autophagy and apoptosis in T-DM1 treatment. Autophagy induced by T-DM1 functioned as a cytotoxic mechanism and facilitated apoptosis of breast cancer cells. Mechanistically, the activation of Akt/mTOR pathway was inhibited in T-DM1-mediated autophagy. Thus, our study highlighted a novel anti-tumor mechanism of T-DM1 therapy, and it might provide novel idea for second-generation HER2-targeted ADC.

## Data Availability

All data are fully available.

## Abbreviations

ADC: Antibody–drug conjugate
ADCC: Antibody-dependent cell-mediated cytotoxicity
CCK-8: Cell counting kit-8
CQ: Chloroquine
DM1: Emtansine
HER2: Human epidermal growth factor receptor 2
P70s6K: Protein S6 kinase
T-DM1: Trastuzumab emtansine
TEM: Transmission electron microscopy
4EBP1: 4E-binding protein 1
